# Frailty index and type 2 diabetes with renal complications: insights from Mendelian randomization and retrospective observational study

**DOI:** 10.1080/0886022X.2026.2687230

**Published:** 2026-06-23

**Authors:** Huanhua Wu, Jiaxin Lin, Xiongbin Wu, Xiaozheng Cao, Biao Wu, Hongmei Liu

**Affiliations:** a Central Laboratory, The Affiliated Shunde Hospital of Jinan University, Foshan, Guangdong Province, PR China; b Department of Nephrology, The Affiliated Shunde Hospital of Jinan University, Foshan, Guangdong Province, PR China; c Department of Nuclear Medicine and PET/CT-MRI Center, The First Affiliated Hospital of Jinan University & Institute of Molecular and Functional Imaging, Jinan University, Guangzhou, Guangdong Province, PR China; d Department of Clinical Nutrition, The Affiliated Shunde Hospital of Jinan University, Foshan, Guangdong Province, PR China

**Keywords:** Frailty, Diabetic kidney disease, Mendelian randomization, Machine learning, Neuromuscular dysfunction

## Abstract

**Objectives:**

To investigate the relationship between frailty and diabetic kidney disease (DKD)-related renal complications using Mendelian randomization (MR) and complementary clinical analysis.

**Methods:**

Two-sample MR was performed using genome-wide association study summary statistics to assess the association between genetically predicted frailty and type 2 diabetes with renal complications. Functional annotation and sensitivity analyses, including exclusion of potentially pleiotropic variants and multivariable MR, were conducted. A retrospective hemodialysis cohort (*n* = 100) was analyzed to characterize frailty burden in patients with DKD-related versus non-DKD end-stage renal disease.

**Results:**

Genetically predicted frailty was significantly associated with increased risk of type 2 diabetes with renal complications (inverse-variance weighted odds ratio = 5.30, 95% confidence interval: 1.89–14.91, *p* = 0.002), without substantial pleiotropy or heterogeneity. Functional annotation suggested exploratory neuromuscular and cytoskeletal signals. In the clinical cohort, patients with DKD had higher frailty scores than those without DKD (median 2.0 vs. 1.0, *p* = 0.003). Exploratory machine learning analysis identified dialysis vintage, grip strength, and physical activity as major contributors to DKD classification.

**Conclusions:**

Frailty was associated with DKD-related renal complications, and forward MR suggested a potential causal contribution. Frailty may represent a clinically relevant marker of systemic vulnerability in this setting, although further validation in larger prospective cohorts is needed.

## HIGHLIGHTS

- This study integrates Mendelian randomization with real-world clinical analysis to examine the relationship between frailty and DKD-related renal complications.
- MR analyses suggest a potential causal contribution of frailty, while functional annotation implicates neuromuscular and cytoskeletal processes as exploratory biological signals.
- In the clinical cohort, frailty burden was greater in patients with DKD-related ESRD, and exploratory analyses identified dialysis burden and impaired physical function as relevant correlates.

## Introduction

Diabetic kidney disease (DKD) is one of the most common and serious complications of diabetes mellitus, affecting approximately 40% of patients with diabetes and remaining the leading cause of end-stage renal disease (ESRD) worldwide [1]. Progressive renal impairment in DKD is associated with increased mortality, reduced quality of life, and substantial healthcare burden [2]. In parallel, frailty, a multidimensional syndrome characterized by decreased physiological reserve and heightened vulnerability to stressors, is increasingly recognized as a major clinical concern in populations with chronic kidney disease [3–5].

Accumulating evidence suggests that frailty and DKD may be biologically interconnected. Proposed shared mechanisms include chronic low-grade inflammation, mitochondrial dysfunction, sarcopenia-related metabolic disturbances, and neuromuscular impairment [6,7]. The emerging concept of renal frailty may help bridge general frailty biology with kidney-specific pathophysiology. In this context, frailty reflects not only systemic vulnerability but also the cumulative burden of chronic renal dysfunction, metabolic stress, oxidative injury, and impaired repair capacity. These mechanisms may synergistically contribute to endothelial dysfunction, profibrotic signaling, and mitochondrial injury within glomerular and tubular structures, thereby accelerating DKD progression while also exacerbating frailty-related decline. Frailty has also been associated with worse outcomes in patients with renal dysfunction, raising the possibility that it may represent more than a simple consequence of chronic disease burden [8].

However, the nature and direction of the relationship between frailty and DKD remain incompletely understood. Frailty is not unique to DKD and is widely observed across chronic diseases; however, its interaction with DKD may be of particular interest because diabetic renal complications arise in a setting of chronic metabolic stress, vascular injury, inflammation, and reduced physiological reserve. In this context, it remains unclear whether frailty contributes directly to DKD pathogenesis or primarily reflects advanced metabolic and renal deterioration. Rather than serving as a substitute for conventional markers such as serum creatinine or estimated glomerular filtration rate, frailty may capture broader systemic vulnerability, reduced functional reserve, multisystem decline, and patient-level heterogeneity that are not fully reflected by renal biochemical indices alone. Given the heterogeneous, deficit-accumulation nature of the frailty index, the enrichment results are better viewed as hypothesis-generating clues that may reflect broader multisystem morbidity and functional decline, rather than definitive evidence for a specific neuromuscular–renal mechanism.

This question is difficult to resolve using conventional observational studies alone, as such designs are prone to residual confounding, selection bias, and reverse causation [9]. Although clinical associations between frailty and renal dysfunction have been reported, robust evidence for causality remains limited. Conversely, genetic approaches may strengthen causal inference but offer less direct insight into the phenotypic burden and clinical relevance of frailty in affected patients.

Therefore, in the present study, we adopted a dual-method framework that integrates Mendelian randomization (MR) with real-world clinical analysis. First, we used two-sample MR to examine whether genetically predicted frailty was associated with DKD-related renal complications while reducing confounding and partially addressing, although not definitively excluding, reverse causation. Second, we examined frailty patterns in a hemodialysis cohort to provide complementary phenotypic characterization of frailty burden and related clinical features in patients with DKD-related versus non-DKD ESRD, and to identify key frailty-associated characteristics using machine learning approaches. We also performed sex-stratified analyses to explore potential sex-specific differences. By combining genetic causal inference with complementary clinical phenotyping, this study aimed to provide a more comprehensive understanding of the role of frailty in DKD-related renal complications.

## Material and methods

### Mendelian randomization

#### Study design and study overview

**Figure 1.**
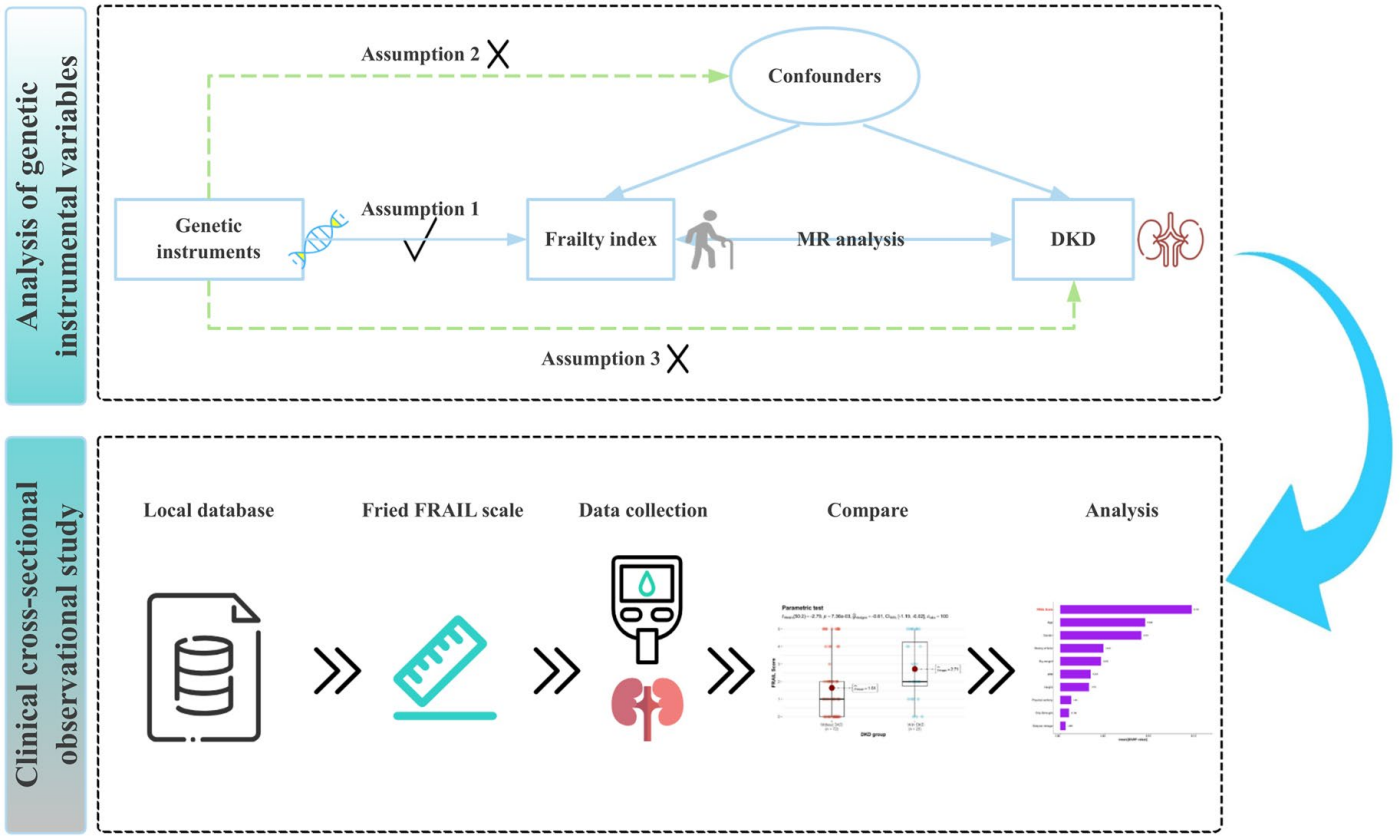
The key assumptions and analytical framework for the MR study investigating the causal relationship between the Frailty index and DKD.

This study used a two-phase analytical framework to investigate the relationship between frailty and DKD (Figure 1). First, we performed two-sample Mendelian randomization (MR) analyses using genome-wide association study (GWAS) summary statistics to assess potential causal effects, focusing on the impact of genetically predicted frailty index on DKD-related renal complications. Second, we examined observational associations between frailty and DKD using clinical data from our local registry. This complementary design enabled comparison of genetic epidemiological findings with real-world clinical patterns. The observational cohort was designed as a cross-sectional comparative analysis in an ESRD population and was not intended to evaluate incident DKD or the longitudinal effect of frailty on earlier-stage DKD development.

### Selection of genetic instruments for MR analyses

To ensure robust causal inference, genetic instrument selection strictly adhered to the three core Mendelian randomization assumptions. Genome-wide significant SNPs (*p* < 5 × 10^−8^) with strong instrument strength (F statistics > 10) were initially selected. Independent variants were obtained through linkage disequilibrium clumping (*r*^2^ < 0.001, window size = 10,000 kb) using European reference data from the 1000 Genomes Project [10]. During harmonization, we excluded SNPs associated with potential confounders or the outcome (*p* < 5 × 10^−8^) and removed palindromic SNPs with intermediate allele frequencies to ensure accurate effect allele matching between exposure and outcome datasets. This comprehensive quality control process minimized biases from weak instruments, linkage disequilibrium, and horizontal pleiotropy while maintaining sufficient genetic variation for reliable causal estimation [11].

### Data sources and genetic instruments selection

Genetic instruments for this MR analysis were derived from two large-scale European-ancestry GWAS datasets available through the IEU OpenGWAS platform (Supplementary Table S1). The exposure dataset (ebi-a-GCST90020053) provided genetic associations with the frailty index [12], whereas the outcome dataset (finn-b-E4_DM2REN) represented type 2 diabetes with renal complications [13]. Because this GWAS phenotype may not be fully identical to conventionally adjudicated clinical DKD, it was treated as a proxy for diabetes-associated renal complication phenotypes. Instrument selection followed a rigorous pipeline implemented. Genome-wide significant SNPs (*p* < 5 × 10^−8^) associated with the frailty index were identified and clumped (r^2^ < 0.001, 10,000 kb window) to ensure independence. Outcome associations were extracted for these instruments while applying quality filters, including imputation quality (r^2^ ≥ 0.8), minor allele frequency thresholds (>1%), and removal of palindromic SNPs with intermediate allele frequencies. The analytical approach incorporated allele alignment and strand orientation checks during data harmonization to ensure the validity of effect estimates. All selected instruments demonstrated sufficient strength (F-statistics >10) to minimize weak instrument bias, with the complete analytical workflow implemented in R, including proper authentication through the OpenGWAS API. To further assess potential pleiotropy, instrumental SNPs were screened using the FastTraitR tool, which was used here as an external SNP–trait look-up resource analogous in purpose to PhenoScanner, to identify reported associations with alternative phenotypes that might indicate pleiotropic pathways.

### Functional annotation

Functional annotation of the final candidate SNPs was conducted using the SNP2GENE workflow implemented in the FUMA platform, which integrates positional, biological, and regulatory information to map genetic variants to genes [14]. Independent significant SNPs were first identified from GWAS summary statistics based on genome-wide significance thresholds and linkage disequilibrium (LD) structure. Lead SNPs and genomic risk loci were subsequently defined by LD clumping, and all SNPs within these loci – including those in LD with lead variants – were systematically annotated for potential biological effects on protein-coding genes, regulatory elements, and chromatin states. To enhance interpretability, these risk loci were cross-referenced against the GWAS Catalog to capture previously reported trait associations.

### Statistical analysis for MR

The MR analyses were performed following established methodological standards to ensure robust causal inference. Primary causal estimates were derived using Inverse variance weighted (IVW) regression with both fixed-effects and multiplicative random-effects models, complemented by other five approaches, including maximum likelihood estimation, weighted median regression, penalized weighted median regression, weighted mode estimation, and IVW radial regression to ensure robust causal inference. To assess potential biases and validate our findings, we conducted comprehensive sensitivity analyses. Heterogeneity was evaluated through forest plots and quantified using Cochran’s Q statistic. Potential pleiotropic effects were systematically examined using: (1) MR-Egger regression to test for directional pleiotropy through intercept analysis, and (2) MR-PRESSO (Pleiotropy RESidual Sum and Outlier) to identify and adjust for outlier variants. The robustness of our results was further verified through leave-one-out sensitivity analyses, with results visualized in corresponding diagnostic plots. All statistical procedures were executed using the TwoSampleMR package (version 0.5.7) in R (version 4.3.1), with effect sizes reported as odds ratios and 95% confidence intervals. Multiple testing correction was applied where appropriate, and statistical significance was evaluated at α = 0.05 (two-tailed).

## Real-world observational analysis

### Patient population

This retrospective cohort study was conducted at The Affiliated Shunde Hospital of Jinan University between January 2023 and January 2025. The observational component was based on retrospective review of existing clinical data and was not a prospective interventional clinical trial. The observational cohort was a feasibility-based/convenience sample determined by the number of consecutive eligible hemodialysis patients with complete clinical and frailty data during the predefined study period, rather than by an *a priori* sample size calculation. From an initial screening of 160 maintenance hemodialysis patients, we established strict inclusion criteria: (1) adult patients (≥18 years) with ESRD confirmed by nephrologists; (2) receiving stable hemodialysis treatment (three sessions weekly) for at least three months; and (3) demonstrating preserved cognitive function for reliable assessment completion. We excluded patients with active malignancies (*n* = 21), decompensated liver cirrhosis (*n* = 18), active systemic infections or autoimmune diseases (*n* = 10), and those with significant communication barriers (*n* = 11).

The study protocol involved comprehensive data collection through electronic medical record review and standardized assessments. Collected parameters included: (1) demographic characteristics (age, sex, dialysis vintage); (2) anthropometric measurements (height, post-dialysis dry weight, body mass index); (3) physical function evaluations (handgrip strength using Jamar dynamometer, documented fall history in previous 12 months); and (4) frailty status determined by the validated 5-item FRAIL scale. For comparative analyses, patients were classified into DKD and non-DKD groups. Although the frailty index used in the MR analysis and the FRAIL scale used in the observational cohort differ in operational definition, both were intended to capture the same underlying construct of frailty from complementary population-level and clinical perspectives.

The Institutional Review Board of The Affiliated Shunde Hospital of Jinan University (No. JDSY-LL-2024187) and granted a waiver of informed consent since all data were collected retrospectively and anonymized prior to analysis.

### Statistical analysis and modeling

Continuous variables were summarized as mean ± standard deviation (SD) or median (interquartile range [IQR]) according to distribution, and categorical variables as counts (percentages). Between-group comparisons were performed using Student’s *t*-test, Wilcoxon rank-sum test, χ^2^ test, or Fisher’s exact test, as appropriate. Sex-stratified analyses were conducted to explore potential sex-specific patterns. To assess whether the association between frailty and DKD was independent of major clinical covariates, multivariable logistic regression was performed with DKD status as the dependent variable and FRAIL score as the primary independent variable, adjusting for age, sex, and dialysis vintage. Given the modest sample size, Firth penalized logistic regression was additionally performed as a sensitivity analysis.

For exploratory machine learning analysis, a support vector machine (SVM) model was developed using the e1071 package in R, with DKD status as the binary outcome. Candidate predictors included demographic, dialysis-related, and functional variables selected according to clinical relevance and data availability. Model interpretability was assessed using SHAP (Shapley Additive Explanations) values calculated with the DALEX package in R. SHAP values were estimated using predict_parts() with type = ‘shap’ and Monte Carlo approximation (*B* = 100), and global feature importance was summarized by mean absolute SHAP values. Sex-stratified SHAP analyses were further performed to explore subgroup-specific predictor patterns.

Given the limited sample size, the machine learning analysis was intended as an exploratory and interpretable approach rather than a formal predictive modeling framework. All analyses were performed in R version 4.3.1 using the ggstatsplot (version 0.13.0), e1071 (version 1.7-16), and DALEX (version 2.5.2) packages. All tests were two-sided, with *p* < 0.05 considered statistically significant.

## Results

### Causal effects of frailty index on DKD by MR

MR analyses provided consistent evidence supporting a positive association between genetically predicted frailty and increased risk of type 2 diabetes with renal complications. Fourteen independent SNPs with adequate instrument strength (mean F-statistic = 39.64, range: 30.0–119.1; Supplementary Table S2) were used as genetic instruments. Across multiple complementary MR approaches, statistically significant associations were observed (Table 1). The primary IVW estimate indicated that each standard deviation increase in genetically predicted frailty index conferred a 5.30-fold higher risk of DKD (95% CI: 1.89–14.91, *p* = 0.002). Consistent results were obtained from maximum likelihood (OR: 5.63, 95% CI: 1.96–16.17, *p* = 0.001), weighted median (OR: 7.24, 95% CI: 1.83–28.73, *p* = 0.005), penalized weighted median (OR: 7.24, 95% CI: 1.86–28.14, *p* = 0.004), and IVW radial regression (OR: 5.31, 95% CI: 2.19–12.85, *p* = 0.001), while the weighted mode method yielded a directionally similar effect with borderline significance (OR: 9.10, 95% CI: 1.26–65.59, *p* = 0.047).

**Table 1.** Causal effects of frailty index on Type 2 diabetes with renal complications outcome estimated by 8 methods.

Exposure	Outcome	Method	nSNP	OR (95% CI)	*p* value
Frailty index	Type 2 diabetes with renal complications	Maximum likelihood	14	5.63 (1.96–16.17)	0.001
Frailty index	Type 2 diabetes with renal complications	Weighted median	14	7.24 (1.83–28.73)	0.005
Frailty index	Type 2 diabetes with renal complications	Penalized weighted median	14	7.24 (1.86–28.14)	0.004
Frailty index	Type 2 diabetes with renal complications	IVW	14	5.30 (1.89–14.91)	0.002
Frailty index	Type 2 diabetes with renal complications	IVW (multiplicative random effects)	14	5.30 (2.19–12.84)	0.001
Frailty index	Type 2 diabetes with renal complications	IVW (fixed effects)	14	5.30 (1.89–14.91)	0.002
Frailty index	Type 2 diabetes with renal complications	Weighted mode	14	9.10 (1.26–65.59)	0.047
Frailty index	Type 2 diabetes with renal complications	IVW radial	14	5.31 (2.19–12.85)	0.001

nSNP: number of single-nucleotide polymorphisms; OR: odds ratio; IVW: inverse variance weighted.

**Figure 2.**
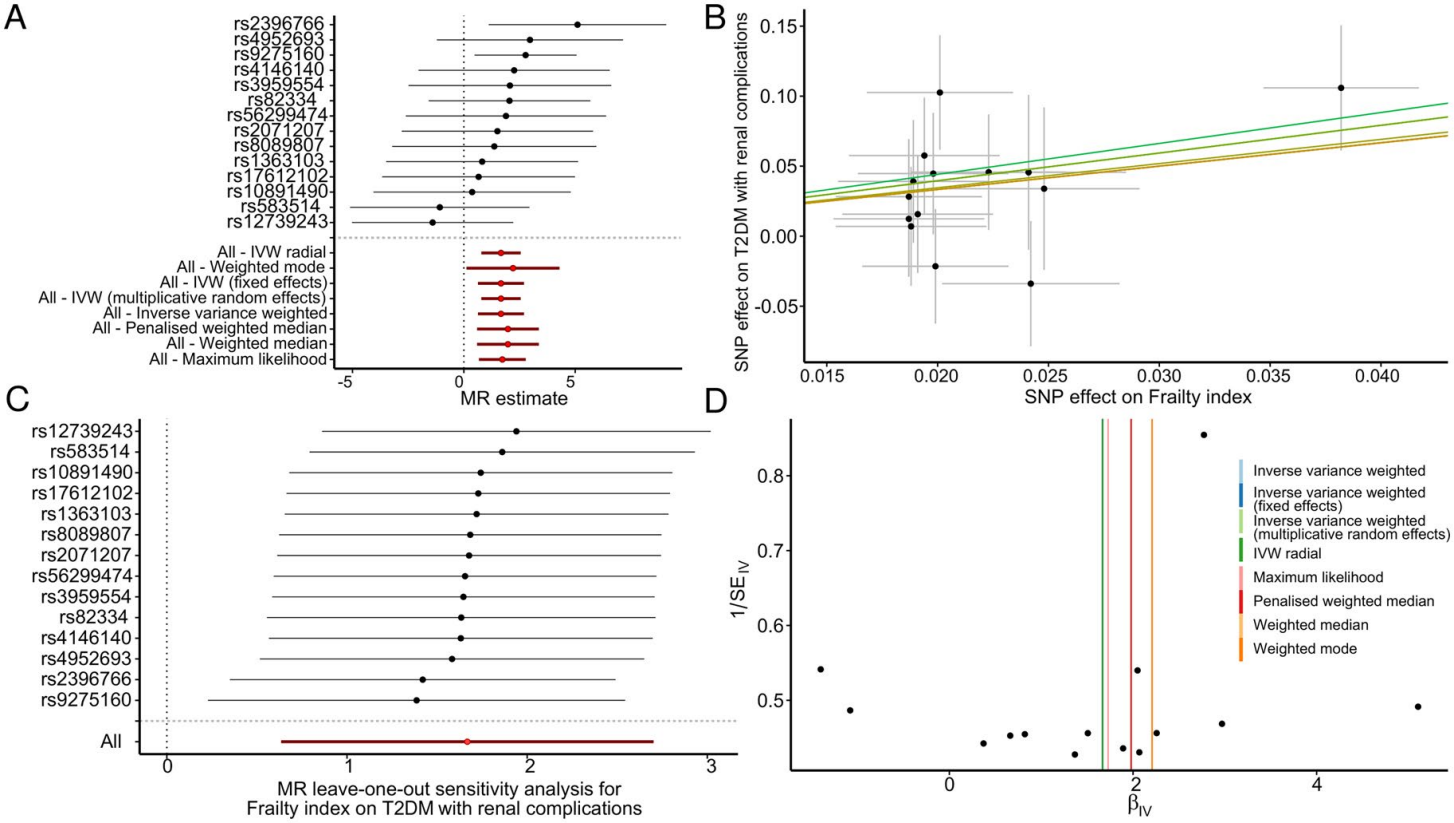
MR analysis of the causal effect of Frailty index on DKD. (A) Forest plot displaying causal estimates for the association between genetically predicted Frailty Index and DKD risk using eight MR methods. The x-axis shows odds ratios (ORs) with 95% confidence intervals, while the y-axis lists individual SNPs and combined estimates. Red lines highlight primary estimates from IVW and other robust methods. (B) Scatter plot of SNP-exposure (Frailty index) effects against SNP-outcome (DKD) effects. Regression lines (colored by method) illustrate the causal relationship, with slope steepness reflecting effect magnitude. The dashed line represents the null hypothesis. (C) Leave-one-out sensitivity analysis demonstrating consistent causal estimates when sequentially excluding each SNP, supporting result robustness. The horizontal line indicates the full-analysis IVW estimate for reference. (D) Funnel plot assessing potential pleiotropy or heterogeneity by plotting SNP effect sizes (x-axis) against precision (1/SE, y-axis). Symmetrical distribution around the IVW estimate (vertical line) suggests minimal bias.

Diagnostic analyses supported the robustness of these findings. As shown in Figure 2(A–B), regression slopes across different MR methods consistently demonstrated a positive association, and the scatter plot revealed a dose–response pattern between SNP effects on frailty and DKD. Leave-one-out analysis (Figure 2(C)) confirmed that exclusion of any single SNP did not materially alter the overall estimates. The funnel plot (Figure 2(D)) exhibited symmetry around the IVW estimate, indicating minimal small-study bias. Sensitivity analyses supported the robustness of the MR findings. No significant heterogeneity was detected (Cochran’s *Q* = 9.52, *p* = 0.732), and neither the MR-Egger intercept (*p* = 0.490) nor the MR-PRESSO global test (*p* = 0.725) suggested directional pleiotropy (Supplementary Table S3). After excluding three potentially pleiotropic SNPs identified by FastTraitR (rs12739243, rs9275160, and rs1363103), the association remained directionally consistent and statistically significant (IVW OR = 5.94, 95% CI: 1.66–21.24, *p* = 0.006), with non-significant MR-Egger intercept and MR-PRESSO global tests (Supplementary Table S4). In multivariable MR adjusting for body mass index and smoking dependency, the frailty-related estimate was attenuated compared with the primary IVW analysis but remained statistically significant (OR = 2.97, 95% CI: 1.23–7.17, *p* = 0.015), suggesting partial overlap with correlated exposures rather than complete explanation by these factors alone (Supplementary Table S5). A matched reverse-direction MR analysis using the DKD-related phenotype could not be performed because valid instrumental variables were not available from the selected GWAS dataset. Therefore, the current directional inference is based primarily on the forward MR analysis.

### Functional mapping and annotation analysis

To further explore the biological mechanisms underlying the observed causal relationship, we performed functional mapping and annotation of frailty-associated genetic variants using the FUMA platform. A total of 11 instrumental SNPs were mapped to multiple genomic loci and annotated to 29 protein-coding genes, including functionally relevant candidates such as SEMA3F, MAPK6, FOXP2, NCAM1, TMOD2, and MST1R (Supplementary Table S6). These genes were distributed across several chromosomal regions, highlighting both neuronal and muscular pathways as potential mediators of the frailty–DKD association.

**Figure 3.**
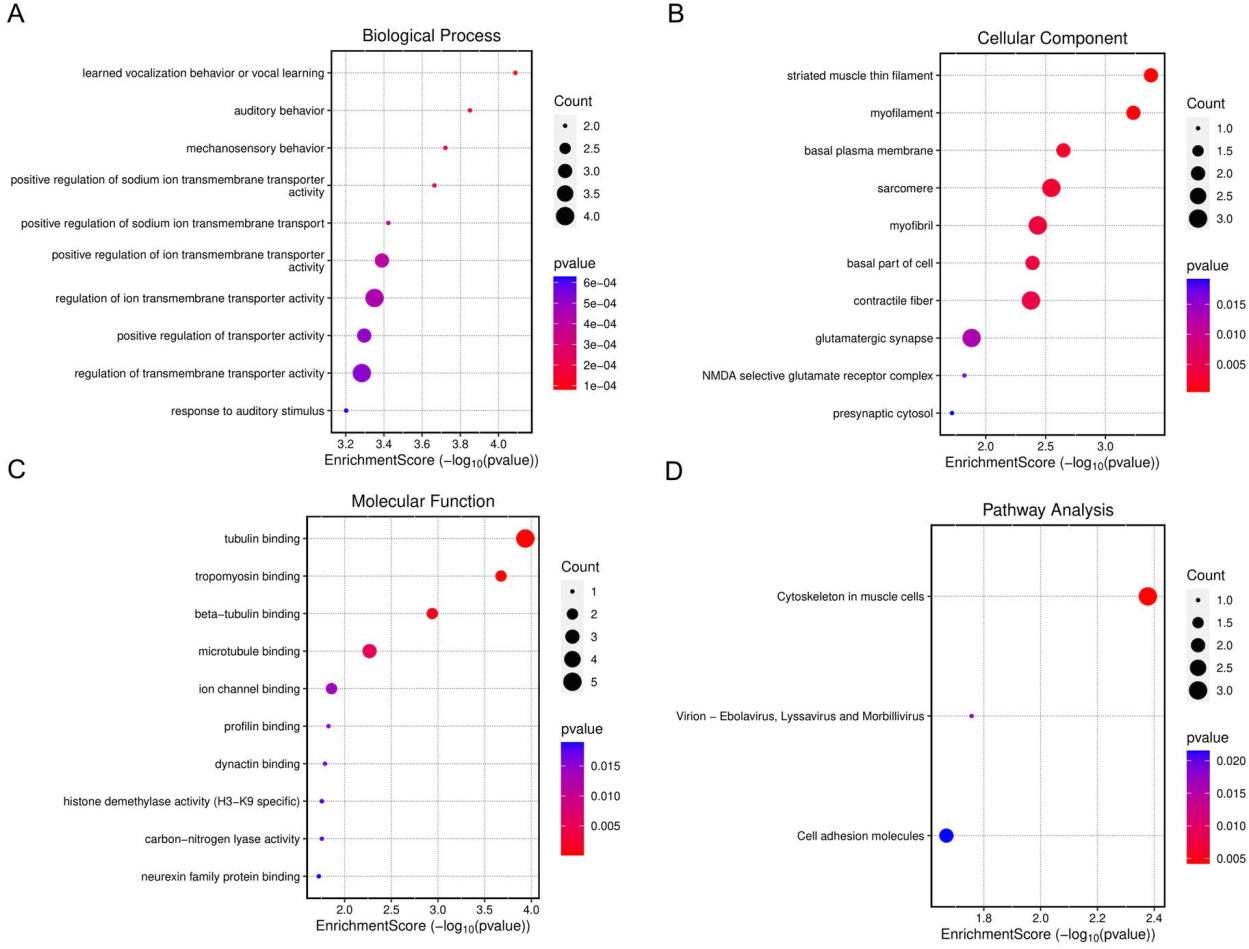
Functional enrichment analysis of biological pathways potentially linking frailty index and DKD. (A) Biological process and molecular function enrichment analysis highlighting cytoskeletal binding functions, including microtubule binding and tubulin binding, as well as synapse-related protein interactions such as neurexin binding. (B) Cellular component enrichment analysis showing significant involvement of muscle-related structures, including myofilament and sarcomere (*p* = 6 × 10^−4^), together with neuronal components such as the glutamatergic synapse. (C) Pathway enrichment analysis identifying three main functional categories: muscle cytoskeleton organization (Enrichment Score = 2.4), viral infection-related pathways (Ebolavirus and Morbillivirus), and cell adhesion molecules (CAMs). (D) Visualization of the statistical significance of enriched component terms, with *p* values ranging from 6 × 10^−4^ to 1 × 10^−4^, supporting the potential role of neuromuscular and cytoskeletal dysfunction in the shared biological mechanisms underlying frailty and DKD.

Functional mapping and annotation analyses suggested enrichment in neuromuscular- and cytoskeleton-related categories (Figure 3(A–B)). Enriched biological process and molecular function terms included cytoskeletal binding functions, such as microtubule and tubulin binding, and synapse-related protein interactions, including neurexin family protein binding. Cellular component enrichment highlighted muscle-related structures, including myofilament and sarcomere (adjusted *p* = 6 × 10^−4^), as well as neuronal compartments such as the glutamatergic synapse.

At the pathway level (Figure 3(C)), enrichment analysis suggested several overrepresented functional categories, including cytoskeletal organization in muscle cells, cell adhesion molecules (CAMs), and certain virus-related pathways. The statistical significance of these terms, visualized by the gradient of adjusted p values (Figure 3(D); Supplementary Figure S1), supports the presence of potentially relevant functional signals. In addition, FUMA locus plots (Supplementary Figure S2) highlighted multiple independent genome-wide significant loci harboring genes implicated in cytoskeletal regulation and neuronal signaling. These enrichment signals should be interpreted as exploratory functional patterns and do not establish a specific biological pathway linking frailty to DKD-related renal complications.

### Observational study

Baseline characteristics are summarized in Table 2. Among 100 hemodialysis patients (28 with DKD, 72 without), those with DKD were older (61.5 ± 7.7 vs. 55.6 ± 15.5 years, *p* = 0.013) and had higher FRAIL scores (median 2.0 [IQR 1.75–4.25] vs. 1.0 [IQR 0.0–2.0], *p* = 0.003). A greater proportion were male (71.4% vs. 48.6%), though not statistically significant (*p* = 0.066). Other characteristics showed no group differences. Because the DKD group was significantly older than the non-DKD group, we further performed multivariable logistic regression to assess whether the association between frailty and DKD was independent of major clinical covariates. After adjustment for age, sex, and dialysis vintage, a higher FRAIL score remained significantly associated with DKD (adjusted OR = 1.41, 95% CI: 1.07–1.90, *p* = 0.016). Sensitivity analysis using Firth penalized logistic regression yielded similar results (adjusted OR = 1.38, 95% CI: 1.06–1.82, *p* = 0.016), supporting the robustness of the observed association despite the modest sample size (Supplementary Table S7).

**Table 2.** Clinical characteristics of 100 patients included.

Variable^a^	Total (*n* = 100)	Without DKD (*n* = 72)	With DKD (*n* = 28)	*p* value^b^
FRAIL Score, (Median [IQR])	2.00 [0.00;3.25]	1.00 [0.00;2.00]	2.00 [1.75;4.25]	0.003
Sex, *n* (%)				0.066
Female	45 (45.00%)	37 (51.39%)	8 (28.57%)	
Male	55 (55.00%)	35 (48.61%)	20 (71.43%)	
Age, year, (Mean ± SD)	57.23 ± 13.97	55.57 ± 15.48	61.50 ± 7.69	0.013
Dialysis vintage age, year, (Median [IQR])	3.00 [1.00;5.00]	3.00 [1.00;5.00]	2.50 [1.75;3.25]	0.509
Height, cm, (Mean ± SD)	161.32 ± 8.60	160.65 ± 9.12	163.04 ± 6.97	0.165
Dry weight, kg, (Mean ± SD)	60.35 ± 10.81	59.67 ± 11.77	62.10 ± 7.74	0.231
BMI, kg/m^2^, (Mean ± SD)	23.08 ± 3.33	22.98 ± 3.74	23.32 ± 1.95	0.554
Grip Strength, kg, (Mean ± SD)	22.25 ± 7.83	22.60 ± 7.91	21.35 ± 7.70	0.472
History of falls, *n* (%)				0.216
No	58 (58.00%)	45 (62.50%)	13 (46.43%)	
Yes	42 (42.00%)	27 (37.50%)	15 (53.57%)	
Physical activity, *n* (%)				0.342
No	66 (66.00%)	45 (62.50%)	21 (75.00%)	
Yes	34 (34.00%)	27 (37.50%)	7 (25.00%)	

^a^ Continuous variables were presented as mean ± SD for normal distribution or median (IQR) for non-normal distribution and categorical variables as Number (%).

^b^ *p* values were calculated using the Student’s t-test for normally distributed or Wilcoxon rank-sum test for non-normally distributed continuous variables and the χ^2^ test for categorical variables.

SD: standard deviation; IQR: interquartile range.

**Figure 4.**
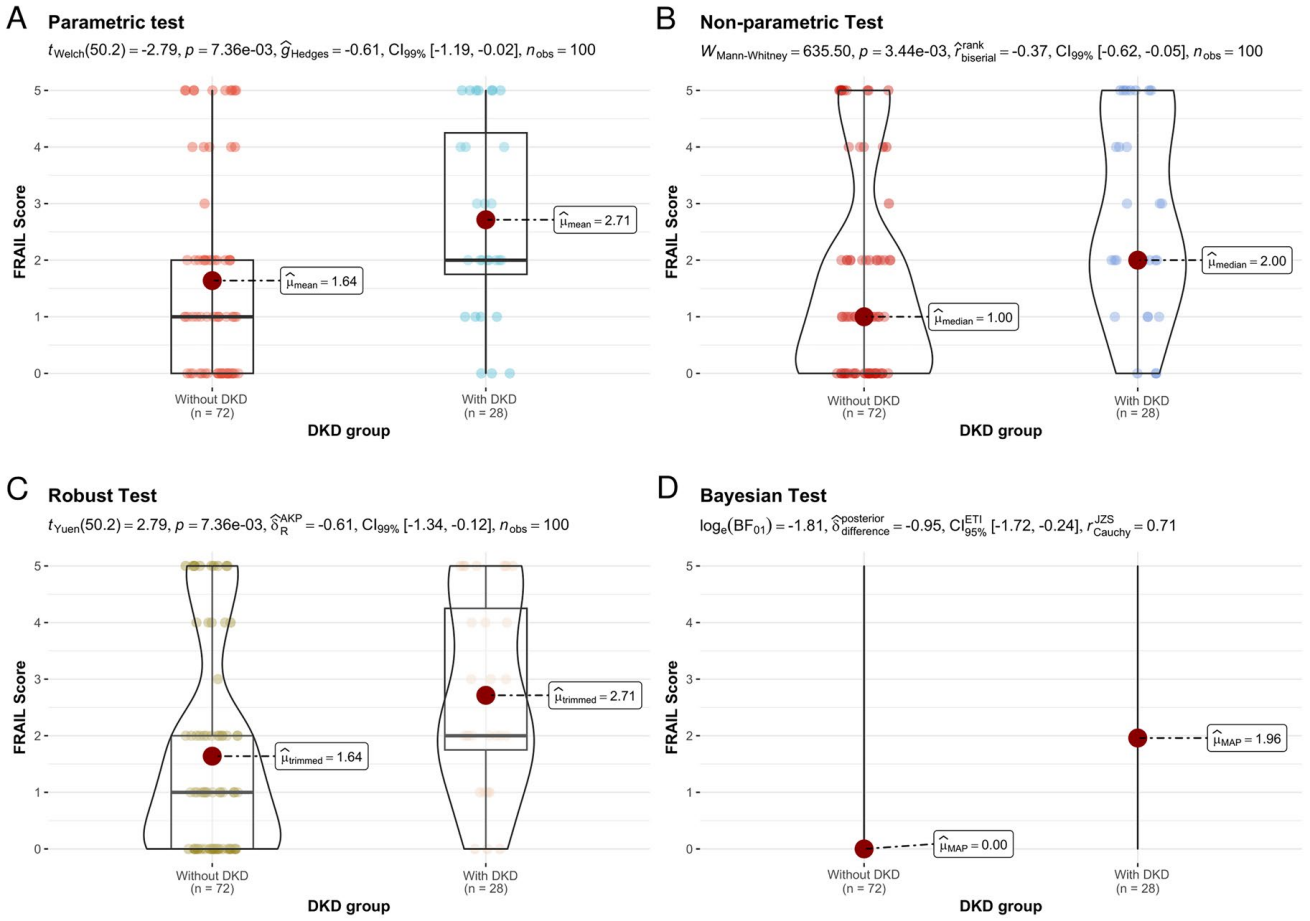
Statistical comparisons of frailty and clinical characteristics between DKD and Non-DKD groups. (A) Parametric analysis (Welch’s *t*-test): Significant group difference in frailty scores (*t* = 2.79, *p* = 7.36 × 10^−^³). (B) Non-parametric analysis (Mann-Whitney U test): Robust confirmation of group differences (*W* = 635.50, *p* = 3.44 × 10^−^³). (C) Robust statistical analysis (Yuen’s *t*-test): Trimmed mean comparison (*t* = 2.79, *p* = 7.36 × 10^−^³). (D) Bayesian analysis: Strong evidence against null (log_e_BF_01_ = −1.81).

Comparisons of frailty burden between groups are shown in Figure 4. Parametric testing using Welch’s t-test confirmed greater frailty in the DKD group (*t* = 2.79, *p* = 7.36 × 10^−^³; Figure 4(A)). This finding was corroborated by non-parametric Mann–Whitney U tests (*W* = 635.50, *p* = 3.44 × 10^−^³; Figure 4(B)) and robust Yuen’s t-tests with trimmed means (*t* = 2.79, *p* = 7.36 × 10^−^³; Figure 4(C)). Bayesian analysis also provided strong evidence against the null hypothesis (logₑBF_01_ = −1.81; Figure 4(D)), collectively demonstrating consistent group-level differences in frailty. Sex-stratified analyses confirmed consistent patterns across sex (Supplementary Figure S3–S4). In both female (*n* = 45) and male patients (*n* = 55), frailty scores were significantly higher in the DKD group, as supported by parametric, non-parametric, robust, and Bayesian tests (all *p* < 0.05). These findings underscore the robustness of the frailty–DKD association across sex.

**Figure 5.**
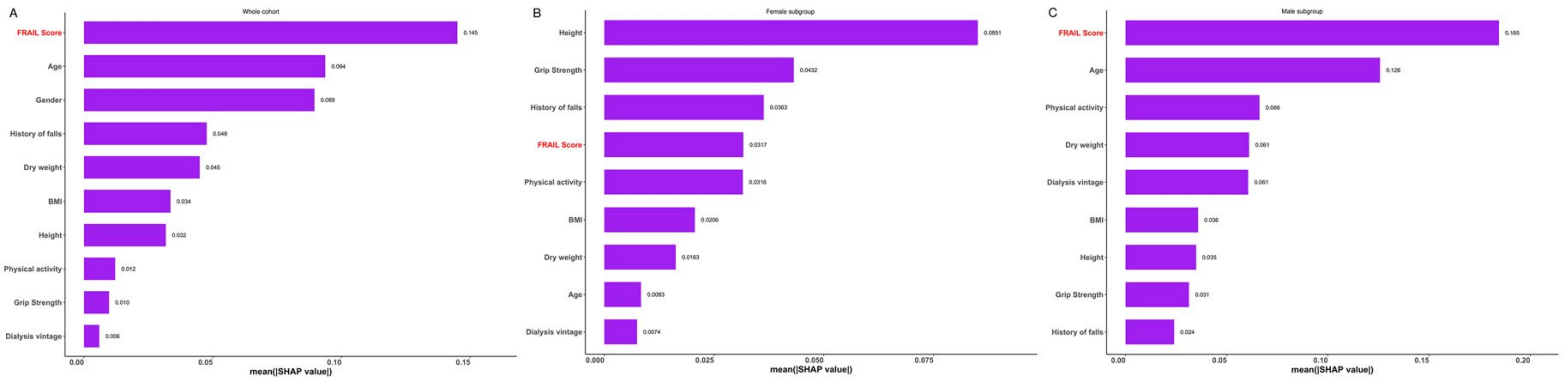
SHAP-based interpretation of clinical variables contributing to SVM classification of DKD. (A) Whole cohort: SHAP analysis of the SVM model identified dialysis vintage as the most influential contributor to DKD classification (mean |SHAP| = 0.145), followed by grip strength (0.094) and physical activity (0.089). (B) Female subgroup: In women, dialysis vintage remained the dominant contributor (0.085), followed by age (0.043) and physical activity (0.032). (C) Male subgroup: In men, history of falls showed the strongest contribution (0.185), followed by grip strength (0.126).

To further explore the clinical variables contributing to DKD classification, we applied a support vector machine (SVM) model with SHAP-based interpretation (Figure 5). In the overall cohort, dialysis vintage emerged as the most influential contributor to model-based DKD classification (mean |SHAP| = 0.145), followed by grip strength (0.094) and physical activity (0.089). In contrast, the FRAIL score showed a relatively modest contribution (0.010), comparable to that of age (0.010) (Figure 5(A)). Sex-stratified analyses suggested distinct predictor patterns. In women, dialysis vintage remained the dominant contributor (0.085), followed by age (0.043) and physical activity (0.032), whereas the FRAIL score contributed less substantially (0.007) (Figure 5(B)). In men, a history of falls showed the strongest contribution (0.185), followed by grip strength (0.126), and the contribution of the FRAIL score was greater than that observed in women (0.024 vs. 0.007) (Figure 5(C)). Given the exploratory nature of this analysis, the absence of formal predictive benchmarking, and the limited subgroup sample sizes, these sex-specific patterns should be interpreted cautiously as hypothesis-generating rather than definitive. These clinical findings should be interpreted as descriptive comparisons in an advanced ESRD population rather than evidence regarding DKD onset or clinical causality.

## Discussion

In this study, we integrated genetic epidemiology with real-world clinical data to investigate the causal relationship between frailty and DKD-related renal complications. The forward MR analyses consistently supported a positive association between genetically predicted frailty and the outcome across multiple complementary methods, and this direction was generally preserved in sensitivity analyses addressing pleiotropy and heterogeneity. However, the magnitude of the effect estimates should be interpreted cautiously. In particular, the attenuation of the frailty-related estimate from OR 5.30 in the primary IVW analysis to OR 2.97 after multivariable adjustment for body mass index and smoking dependency suggests that correlated exposures contribute substantively to the observed association. Accordingly, our findings are more informative for the direction and robustness of the frailty-related signal than for the precise magnitude of effect. Functional annotation further highlighted neuromuscular and cytoskeletal pathways as potential biological mediators [15,16]. Complementary observational data from a well-characterized hemodialysis cohort confirmed that patients with DKD exhibit a substantially higher frailty burden, with consistent associations across parametric, non-parametric, robust, and Bayesian statistical frameworks. Importantly, sex-stratified analyses revealed sex-specific patterns, underscoring the heterogeneity of frailty–DKD interactions in clinical practice.

Our findings advance understanding of the frailty–DKD relationship by addressing, at least in part, a major limitation of prior observational studies, namely reverse causation. Although frailty has long been associated with adverse outcomes in chronic kidney disease [17–19], whether it acts as a causal contributor or merely reflects progressive renal decline has remained uncertain. Using Mendelian randomization, we found evidence supporting a positive association between genetically predicted frailty and DKD-related renal complications [20]. Importantly, this does not imply that frailty is uniquely specific to DKD or that it should replace conventional renal markers such as serum creatinine or estimated glomerular filtration rate. Rather, frailty may capture broader systemic vulnerability and reduced functional reserve that are not fully reflected by biochemical indices alone and may be particularly relevant in the context of DKD-related disease burden.

This interpretation remains biologically plausible, given the overlap between frailty and DKD in chronic inflammation, oxidative stress, mitochondrial dysfunction, metabolic disturbance, and impaired repair. These processes may contribute to both renal injury and multisystem functional decline. In this sense, the importance of frailty in DKD may lie not in disease-specific exclusivity, but in the convergence of metabolic, inflammatory, and functional pathways that make frailty especially relevant to renal vulnerability in this setting. The consistency between the MR findings and the clinical observations in our dialysis cohort, therefore, supports the relevance of frailty as a complementary clinical phenotype in advanced DKD-related disease burden and suggests potential value for risk stratification, individualized supportive care, and future mechanistic investigation.

Functional mapping analyses provided an exploratory biological context for the frailty–DKD relationship. Genes mapped to frailty-associated SNPs showed enrichment in cytoskeletal and neuromuscular categories, which may be broadly consistent with musculoskeletal dysfunction and reduced physical reserve [21,22]. However, given the heterogeneous, deficit-accumulation nature of the frailty index, these enrichment signals should be interpreted cautiously and may equally reflect the broader genetic architecture of general morbidity, physical disability, or multisystem functional decline. The enrichment of virus-related pathways is even less specific and may represent broader immune- or stress-response signatures rather than direct DKD-related mechanisms [23–26]. Overall, these annotation results are best viewed as hypothesis-generating clues rather than definitive evidence for a specific biological pathway linking frailty to DKD-related renal complications [27,28].

Our observational analyses should be interpreted as providing complementary phenotypic context rather than evidence regarding whether frailty contributed to DKD development. In this advanced ESRD cohort, patients with DKD had higher frailty scores than those without DKD, independent of the measured clinical covariates, indicating a greater frailty burden in DKD-related disease. In the exploratory machine learning analysis, dialysis vintage, grip strength, and physical activity were the main contributors to SVM-based DKD classification, suggesting that cumulative dialysis burden and impaired functional reserve are important clinical correlates in this population. These observations were broadly consistent with the exploratory genetic annotation signals, but should not be interpreted as confirming a specific mechanism. Rather, reduced grip strength, falls, and longer dialysis vintage may reflect broader multisystem functional impairment and cumulative physiological stress in advanced renal disease. Sex (gender)-stratified analyses further suggested distinct predictor patterns, with age and physical activity more prominent in women and fall history and grip strength more influential in men. However, given the exploratory nature of these analyses, the absence of diabetes-specific covariates, and the limited subgroup sample sizes, these findings should be interpreted cautiously as descriptive and hypothesis-generating rather than confirmatory [29,30].

From a clinical perspective, our findings argue for early frailty screening as an integral part of DKD care. Routine use of simple, validated tools such as the FRAIL scale could facilitate risk stratification and timely interventions, including exercise-based rehabilitation, nutritional optimization, and individualized dialysis regimens. Incorporating frailty assessment into DKD management may also improve prognostic accuracy and guide resource allocation in multidisciplinary nephrology care.

This study has several strengths, including the integration of MR and clinical analyses, rigorous instrument selection, and multiple sensitivity analyses. The dual-method design also enabled complementary evaluation of the frailty–DKD relationship from both causal inference and real-world clinical perspectives. Nevertheless, several limitations should be acknowledged. First, the MR analyses were restricted to European-Ancestry GWAS data, which may limit generalizability to other populations. Second, although the FRAIL score is widely validated, it may not fully capture cognitive and psychosocial dimensions of frailty. Third, the observational component was based on a single-center, feasibility-based sample with a relatively small number of DKD cases, which may have reduced statistical power, limited precision, and restricted generalizability. This was particularly relevant for the sex-stratified analyses, where very small subgroup sizes likely reduced estimate stability; these findings should therefore be considered exploratory. Fourth, residual confounding cannot be excluded, particularly because diabetes-specific variables such as HbA1c and diabetes duration were unavailable. The observational cohort was also intended as a complementary phenotypic component rather than a stand-alone confirmatory dataset and was not designed to address DKD onset or longitudinal disease progression. Fifth, the machine learning analysis was exploratory and interpretable rather than a formal predictive modeling study; therefore, no predefined train/test split, extensive cross-validation, or comprehensive performance benchmarking was performed, and overfitting cannot be excluded, especially in the sex-stratified analyses. Finally, the MR outcome was based on the GWAS phenotype ‘type 2 diabetes with renal complications’ rather than a strictly adjudicated clinical DKD definition and may therefore capture a heterogeneous spectrum of renal pathologies in diabetic patients. Accordingly, the MR findings should be interpreted as relating to diabetes-associated renal complications more broadly rather than to narrowly defined clinical DKD alone. In addition, although the forward MR analysis supported a positive association between genetically predicted frailty and DKD-related renal complications, a matched reverse-direction MR analysis could not be performed because valid instrumental variables for the selected outcome phenotype were unavailable. Thus, the directional inference remains incomplete, and future studies using alternative renal phenotypes such as eGFR, albuminuria, or broader CKD traits will be important to further evaluate reverse-direction hypotheses.

## Conclusions

Our findings provide convergent genetic and clinical evidence supporting the relevance of frailty in DKD-related renal complications, with forward MR analyses suggesting a potential causal contribution of frailty. Exploratory functional annotation identified potentially relevant biological signals, although these findings were nonspecific and should be interpreted cautiously. Overall, these results support frailty as a complementary phenotype of systemic vulnerability in DKD and warrant validation in larger prospective cohorts.

## Supplementary Material

FigureS3.tiff

FigureS4.tiff

FigureS1.tiff

FigureS2.tiff

## Data Availability

The data that support the findings of this study are available from the corresponding author upon reasonable request.

## Clinical trial number

This study was approved by the relevant Institutional Review Boards and registered in the Chinese Clinical Trial Registry (ChiCTR2500107040).

## Clinical relevance statement

Frailty is prevalent in chronic kidney disease and is usually considered a marker of reduced physiological reserve. In this study, genetic and clinical analyses consistently supported the relevance of frailty to DKD-related renal complications. Although not a substitute for conventional renal markers, frailty may provide complementary information on systemic vulnerability and may help inform risk stratification and individualized supportive care in nephrology practice.
