# Supplementary figures and images for: Frailty index and type 2 diabetes with renal complications: insights from Mendelian randomization and retrospective observational study
Source: Ren Fail. 2026 Jun 23;48(1):2687230. doi: 10.1080/0886022X.2026.2687230 (PMC13292309; doi:10.1080/0886022X.2026.2687230)

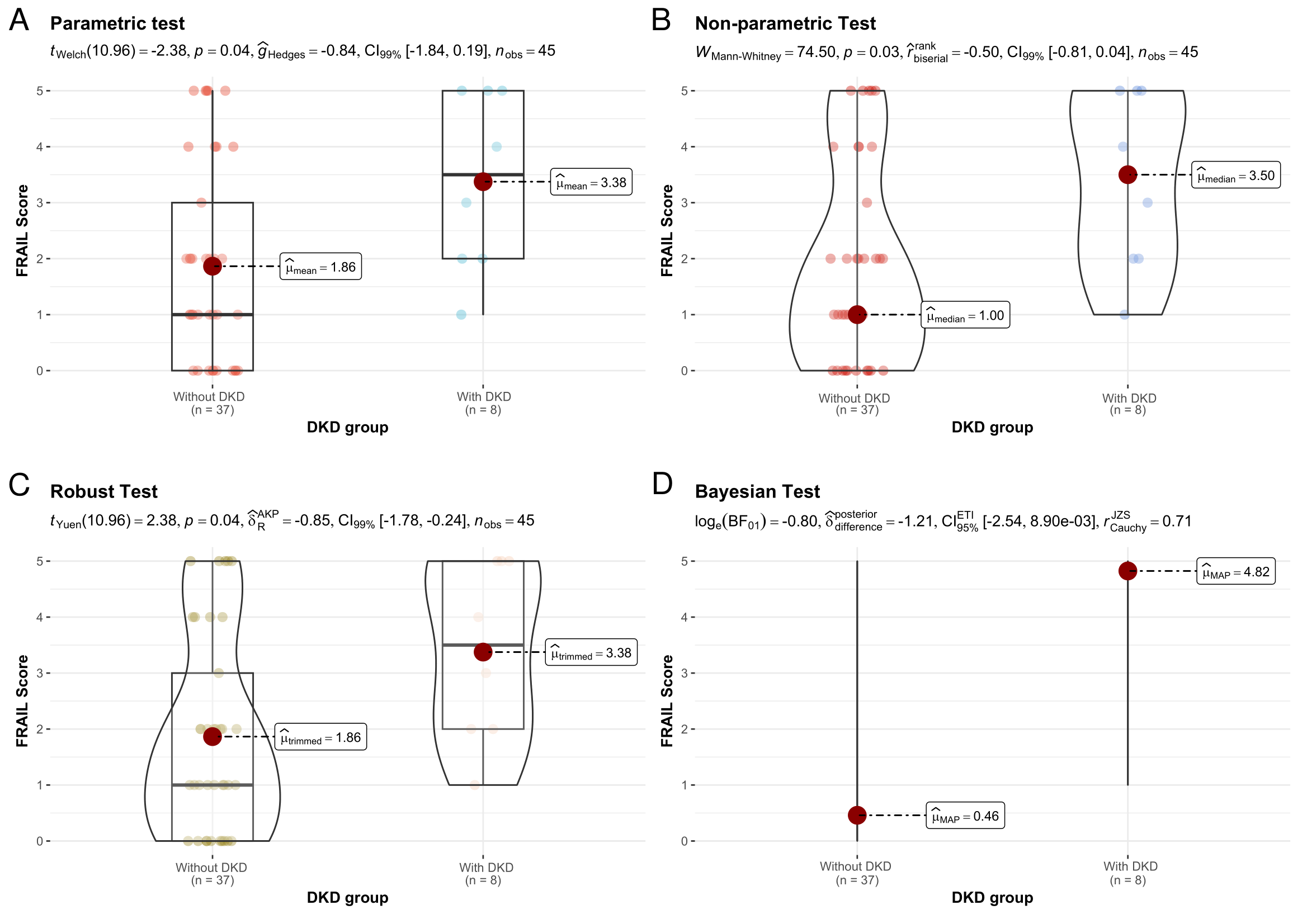

Supplement: FigureS3.tiff [file IRNF_A_2687230_SM4415.tiff]

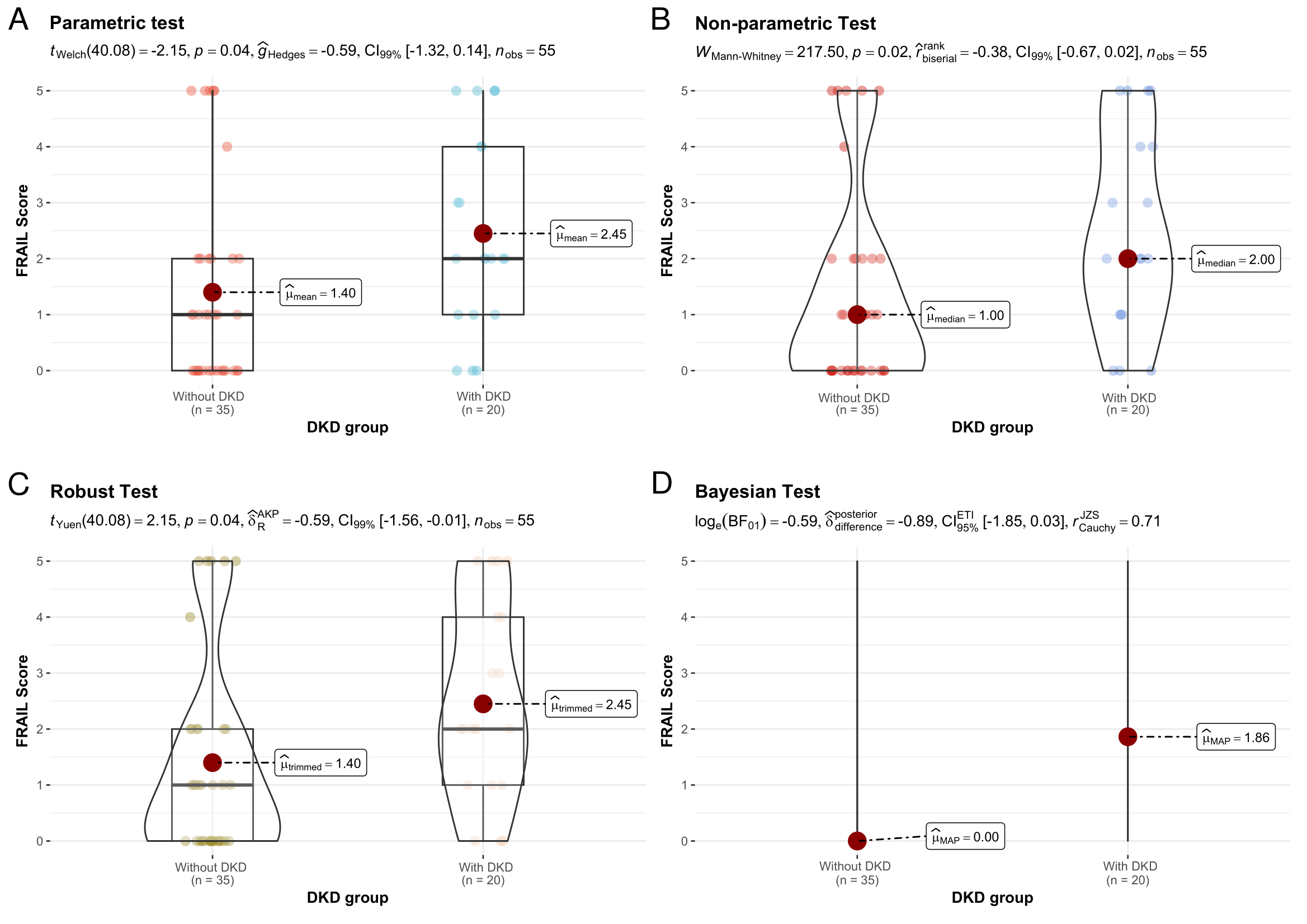

Supplement: FigureS4.tiff [file IRNF_A_2687230_SM4413.tiff]

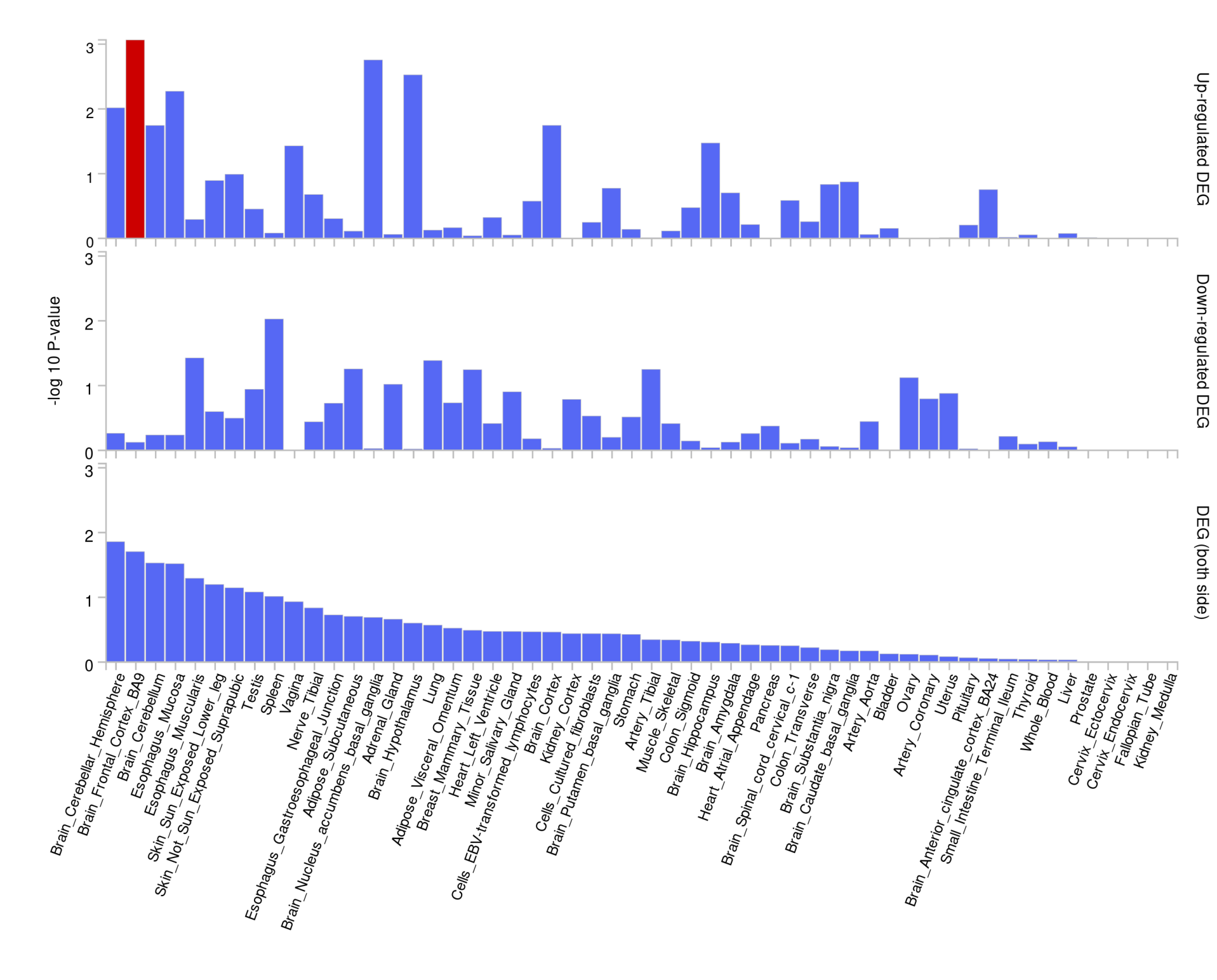

Supplement: FigureS1.tiff [file IRNF_A_2687230_SM4412.tiff]

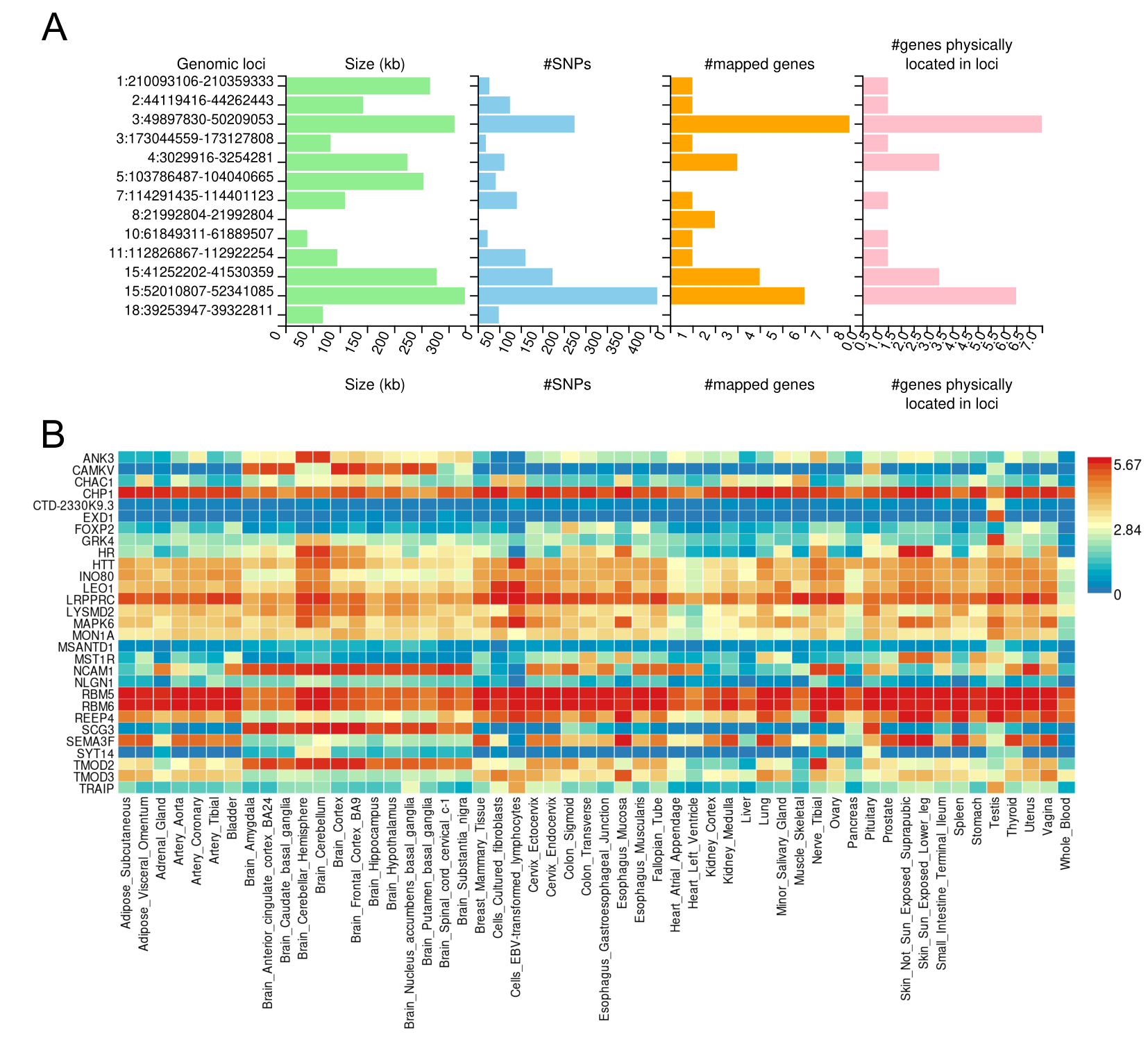

Supplement: FigureS2.tiff [file IRNF_A_2687230_SM4411.tiff]
